# Cross-talks of tumor and myeloid cells regulate the therapeutic responses to chemotherapy by modulating innate immune responses

**DOI:** 10.1186/2051-1426-1-S1-O16

**Published:** 2013-11-07

**Authors:** Masahisa Jinushi, Muhammad Baghdadi, Hideo Yagita

**Affiliations:** 1 Institute for Genetic Medicine, Hokkaido University, Sapporo, Japan; 2 Department of Immunology, Juntendo University, Tokyo, Japan

## 

Tumor-associated myeloid cells play a critical role in supporting tumor progression and metastasis. However, molecular mechanisms by which tumor cells interact with myeloid cells to modify therapeutic responses to anticancer treatments remain largely unknown. We identified several key factors affecting antitumor effects of chemotherapy by manipulating the modes of interaction between tumor cells and myeloid cells within tumor microenvironments. Tumor-derived factors such as VEGF-A and arginase-I upregulate TIM-3 on dendritic cells. TIM-3 interacts with HMGB1 released from chemotherapy-treated tumor cells, and suppresses innate pattern recognition signals mediated by nucleic acids from dying tumor cells. The inhibition of TIM-3 augments antitumor effects of cytotoxic chemotherapy against established tumors. Moreover, danger-associated molecular patterns (DAMPs) released from chemotherapy-damaged tumor cells induced TIM-4 on TAMs recruited from bone marrow-derived precursors. TIM-4 directly interacted with AMPK-α1 at phagosome and activated autophagy-mediated lysosomal degradation of ingested tumors, leading to reduced antigen presentation and impaired CTL responses. Consistently, blockade of the TIM-4/AMPK-α1/ autophagy pathway augmented antitumor effect of chemotherapeutics by enhancing tumor-specific CTL responses. Taken together, our findings provide new evidences that targeting of key factors regulating interplay between tumor cells and myeloid cells provides new therapeutic strategy to eradicate chemoresistant tumors.

**Figure 1:**
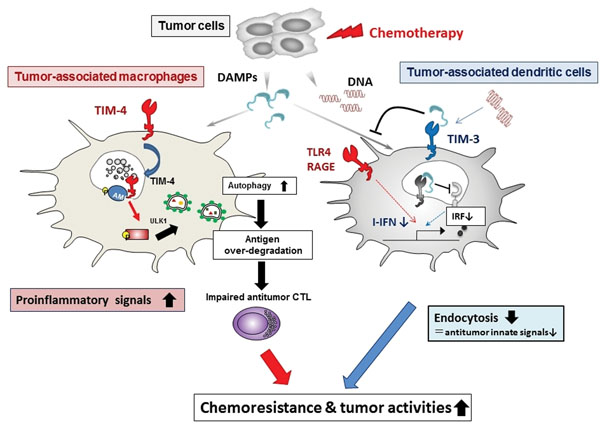
Cross-talk between tumors and myeloid cells suppresses antitumor effect of chemotherapy.

